# Pseudomonas Acts as a Reservoir of Novel Tigecycline Resistance Efflux Pump *tmexC6D6-toprJ1b* and *tmexCD-toprJ* Variants

**DOI:** 10.1128/spectrum.00767-23

**Published:** 2023-04-17

**Authors:** Cheng-Zhen Wang, Xun Gao, Xin-Hong Liang, Lu-Chao Lv, Li-Tao Lu, Chao Yue, Xiao-Xiao Cui, Ke-Er Yang, Duo Lu, Jian-Hua Liu, Jun Yang

**Affiliations:** a Guangdong Provincial Key Laboratory of Veterinary Pharmaceutics Development and Safety Evaluation, Key Laboratory of Zoonosis of Ministry of Agricultural and Rural Affairs, National Risk Assessment Laboratory for Antimicrobial Resistant of Microorganisms in Animals, College of Veterinary Medicine, South China Agricultural University, Guangzhou, China; b Guangdong Laboratory for Lingnan Modern Agriculture, Guangzhou, China; Yangzhou University

**Keywords:** *Pseudomonas*, tigecycline resistance, efflux pump, *tmexCD-toprJ*, transcription regulator, promoter

## Abstract

Several variants of the plasmid-carried tigecycline resistance gene cluster, *tmexCD-toprJ*, have been identified. This study characterized another novel variant, *tmexC6D6-toprJ1b*, located on the chromosome of environmental-origin Pseudomonas mendocina. TMexC6D6-TOprJ1 mediates resistance to multiple drugs, including tigecycline. The promoter activity of *tmexC6D6-toprJ1b* and negative transcriptional repression by the upstream regulator tnfxB6 are crucial for the expression of *tmexC6D6-toprJ1b*. *tmexC6D6-toprJ1b* was found in the plasmids or chromosomes of different Pseudomonas species from six countries. Two genetic backgrounds, class 1 integrons and *int*-carrying integrase units, were found adjacent to the *tmexC6D6-toprJ1b* gene cluster and might mediate the transfer of this novel efflux pump gene cluster in Pseudomonas. Further phylogenetic analysis revealed Pseudomonas as the major reservoir of *tmexCD-toprJ* variants, warranting closer monitoring in the future.

**IMPORTANCE** Tigecycline is one of the treatment options for serious infections caused by multidrug-resistant bacteria, and tigecycline resistance has gained extensive attention. The emergence of a transferable tigecycline resistance efflux pump gene cluster, *tmexCD-toprJ*, severely challenged the efficiency of tigecycline. In this study, we identified another novel *tmexCD-toprJ* variant, *tmexC6D6-toprJ1b*, which could confer resistance to multiple classes of antibiotics, including tigecycline. Although *tmexC6D6-toprJ1b* was found only in Pseudomonas species, *tmexC6D6-toprJ1b* might spread to *Enterobacteriaceae* hosts via mobile genetic elements resembling those of other *tmexCD-toprJ* variants, compromising the therapeutic strategies. Meanwhile, novel transferable *tmexCD-toprJ* variants are constantly emerging and mostly exist in Pseudomonas spp., indicating Pseudomonas as the important hidden reservoir and origin of *tmexCD-toprJ* variants. Continuous monitoring and investigations of *tmexCD-toprJ* are urgent to control its spread.

## OBSERVATION

Tigecycline is one of the few last-resort drugs for the treatment of multidrug-resistant bacterial infections, resistance to which poses a serious threat to public health worldwide (1–3). Recently, plasmid-carried *tet*(X)-degrading enzyme genes and the resistance nodulation division (RND)-type efflux pump gene cluster *tmexCD-toprJ* were reported to mediate tigecycline resistance in Gram-negative bacteria (4, 5). TMexCD-TOprJ efflux pumps not only recognize tigecycline but also expel multiple drugs as efflux substrates, thereby conferring multidrug resistance (5). Since the first report of the *tmexCD-toprJ* gene cluster *tmexCD1-toprJ1* in Klebsiella pneumoniae in 2020, other *tmexCD-toprJ* variants, including *tmexCD2-toprJ2* in Raoultella ornithinolytica and K. pneumoniae, *tmexCD3-toprJ1b* in Proteus and Pseudomonas, *tmexCD4-toprJ4* in Klebsiella quasipneumoniae and Enterobacter roggenkampii, and *tmexC3D5-toprJ2b* in *Oceanimonas*, have been discovered (6–9). *tmexCD1-toprJ1* and *tmexCD3-toprJ1b* are widely detected in clinical settings, animals, and the environment in China, and *tmexCD2-toprJ2* is mostly detected in human isolates (5, 7–13). These *tmexCD-toprJ* variants are located on plasmids or chromosomes, predominantly carried by mobile elements such as integrative and conjugative elements and transposons (5–13). Mobile *tmexCD-toprJ* gene clusters showed approximately 70% nucleotide identity with the chromosomally located RND family efflux pump *mexCD-oprJ*, which is silent under the tight regulation of NfxB in Pseudomonas (5, 7, 14). Therefore, *tmexCD-toprJ* was predicted to have originated from chromosomal ancestral efflux pump genes in Pseudomonas (5, 10). Nevertheless, Pseudomonas can develop tigecycline resistance by acquiring transferable *tmexCD-toprJ* gene clusters, such as *tmexCD3-toprJ1b*, and act as a reservoir for capturing other antimicrobial resistance genes (ARGs) (7, 15). In this study, we characterized the function and genetic features of another novel transferable efflux pump gene cluster, *tmexC6D6-toprJ1b*, on the chromosome of environmental-origin Pseudomonas mendocina. Our results indicate Pseudomonas as the potential major reservoir of *tmexCD-toprJ*, promoting its wide distribution.

In June 2022, isolate GD22SC3150TT was collected from an environmental sample of a farmers’ market in Guangdong province of China using MacConkey agar plates supplemented with 4 mg/L tigecycline. GD22SC3150TT was classified as P. mendocina via matrix-assisted laser desorption ionization–time of flight mass spectrometry (Bruker Daltonics, Bremen, Germany). Moreover, a PCR screening assay confirmed that a *tmexCD-toprJ*-like gene cluster was carried by this strain. Antimicrobial susceptibility testing was performed using the broth or agar dilution method (testing for tigecycline and colistin), according to the Clinical and Laboratory Standards Institute guidelines (16). GD22SC3150TT was found to be resistant to tigecycline (MIC = 8 mg/L), ceftazidime (MIC = 8 mg/L), cefotaxime (MIC = 16 mg/L), cefepime (MIC = 8 mg/L), and ciprofloxacin (MIC > 64 mg/L) but susceptible to colistin and imipenem (Table 1).

**TABLE 1:** Various antibiotic susceptibility profiles (MICs, mg/L) of strains in this study

Strain	Strain information	MIC (mg/L) of drug^*a*^:									
		TIG	MIN	DOX	TET	CQM	CTX	CAZ	FEP	STR	CIP
Pseudomonas mendocina GD22SC3150TT	Isolated from environmental sample	8	16	32	>128	8	16	8	8	>256	>64
Escherichia coli											
DH5α	Recipient strain	0.25	0.5	0.5	0.5	0.03	0.015	0.06	0.015	1	0.001
DH5α+pJN105	Recombinant strain with empty vector	0.25	0.5	0.5	0.5	0.03	0.015	0.06	0.015	1	0.001
DH5α+pJN105-tmexC6D6-toprJ1b-P6	Transformants expressing *tmexC6D6-toprJ1b* in promoter of *tmexC6*	2	4	4	2	0.06	0.03	0.125	0.03	2	0.002
DH5α+pJN105-tmexCD3-toprJ1b-P3	Transformants expressing *tmexC6D6-toprJ1b* in promoter of *tmexC3*	4	8	16	4	0.25	0.06	0.25	0.125	4	0.004
DH5α+pJN105-tmexCD3-toprJ1b-P1	Transformants expressing *tmexC6D6-toprJ1b* in promoter of *tmexC1*	4	8	16	4	0.25	0.06	0.25	0.125	4	0.004
DH5α+pJN105-tnfxB6-tmexC6D6-toprJ1b	Transformants expressing *tnfxB6-tmexC6D6-toprJ1b* with promoter of *tmexC6*	0.5	2	2	1	0.03	0.015	0.125	0.015	1	0.002

a Abbreviations: TIG, tigecycline; MIN, minocycline; DOX, doxycycline; TET, tetracycline; CQM, cefquinome; CTX, cefotaxime; CAZ, ceftazidime; FEP, cefepime; STR, streptomycin; CIP, ciprofloxacin. The colistin and imipenem MICs for GD22SC3150TT were 0.5 mg/L and 0.25 mg/L, respectively.

To determine the genetic location of this *tmexCD-toprJ*-like gene cluster, the complete genomic data of GD22SC3150TT were generated using the Illumina platform and Nanopore MinION, together with Unicycler version 0.4.7 for genome assemblies (17). GD22SC3150TT consisted of a 4,398,051-bp chromosome and a 35,410-bp plasmid. Six known ARGs, namely, determinants conferring resistance to aminoglycosides [*aac(6′)-IIa* and *aadA8*], chloramphenicol (*cmlA1*), quinolone (*qnrVC1*), sulfonamide (*sul1*), and tetracycline [*tet*(G)], were found on the chromosome. Intriguingly, the *tmexCD-toprJ*-like gene cluster was also detected on the chromosome, accompanied by an adjacent *tnfxB*-like regulator gene, which is highly similar to *tnfxB*3. Further comparison of this gene cluster with other *tmexCD-toprJ* variants revealed that these six *tmexCD-toprJ* gene clusters shared 96.84 to 98.47% nucleotide identity (see Table S1 in the supplemental material). In GD22SC3150TT, TMexC shared 96.90 to 98.45% amino acid identity with TMexC1 to TMexC4, and TMexD shared 97.61 to 99.90% amino acid identity with TMexD1 to TMexD5 (Table S1). The nucleotide sequence of the *toprJ* gene in GD22SC3150TT was the same as that of *toprJ1b* in *tnfxB3-tmexCD3-toprJ1b* (Table S1). Therefore, this gene cluster was designated *tnfxB6-tmexC6D6-toprJ1b* with the corresponding proteins being TNfxB6, TMexC6, TMexD6, and TOprJ1, respectively.

**FIG 1:**
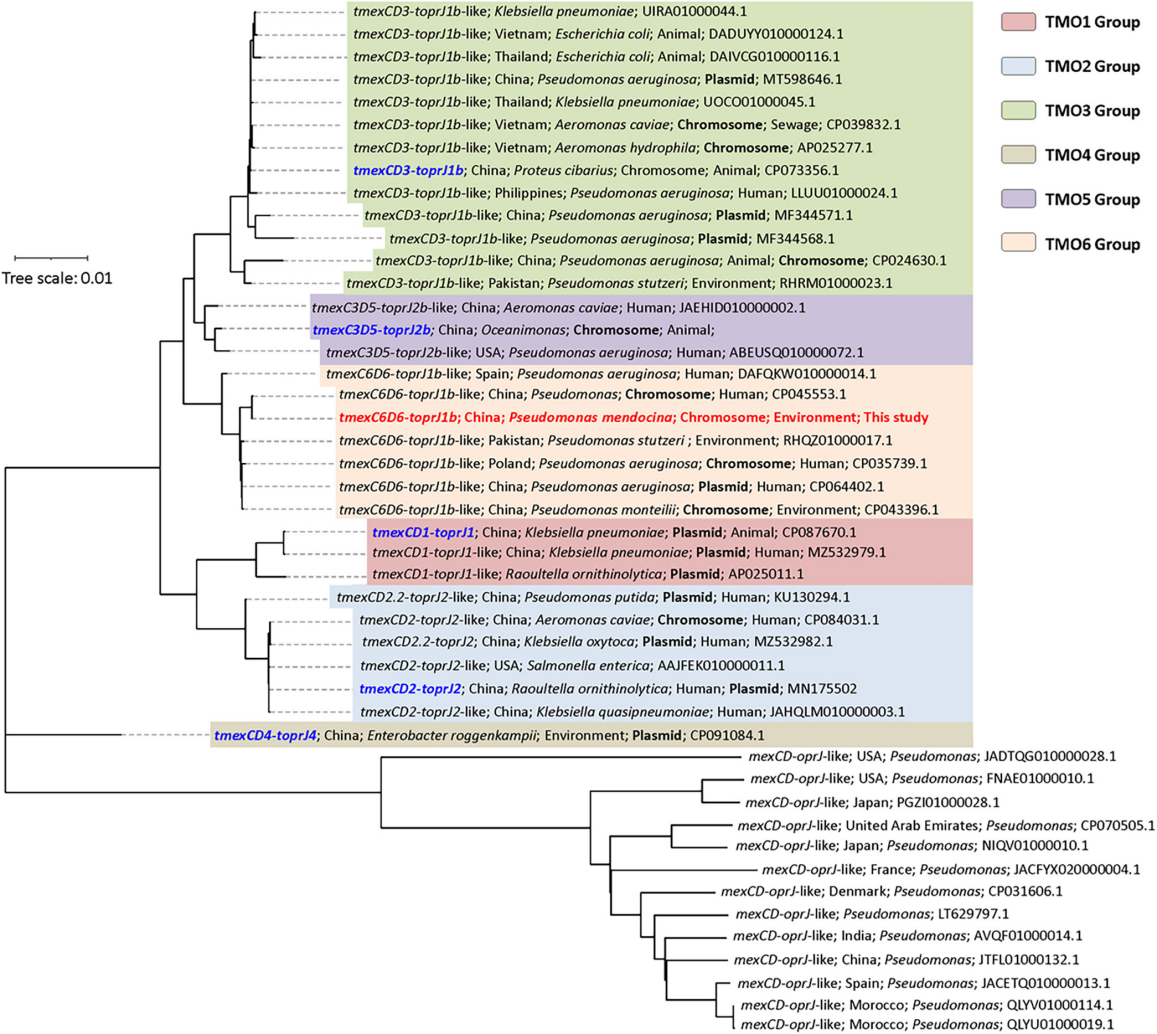
Phylogenetic tree of *tmexC6D6-toprJ1b* gene clusters and its homologs. A total of 46 representative nonredundant nucleotide sequences of *tmexCD1-toprJ1*-like gene clusters from GenBank were selected. This tree was constructed by MEGA-X using the neighbor-joining method with 2,000 replicates. Basic information on these *tmexCD-toprJ*-carrying bacteria, including their GenBank accession numbers, is shown in the corresponding branch. The six background colors indicate the six types of *tmexCD-toprJ* gene clusters.

To fully determine the evolutionary relationship between *tmexC6D6-toprJ1b* and various *tmexCD-toprJ* members, a phylogenetic tree was constructed based on the three protein sequences encoded by *tmexCD-toprJ* using MEGA X and iTOL (Fig. 1) (18, 19). A total of 46 representative nonredundant nucleotide sequences of *tmexCD1-toprJ1*-like gene clusters from GenBank were selected for this tree (Table S2). Seven phylogenetic groups, *tmexCD1-toprJ1*-like (TMO1), *tmexCD2-toprJ2*-like (TMO2), *tmexCD3-toprJ1b*-like (TMO3), *tmexCD4-toprJ4* (TMO4), *tmexC3D5-toprJ2b*-like (TMO5), *tmexC6D6-toprJ1b*-like (TMO6), and *mexCD-oprJ*-like, could be clearly distinguished based on their similarity. The six TMO groups were either accompanied by mobile genetic elements or located in the plasmids, while *mexCD-oprJ*-like gene clusters were all inherent efflux pump gene clusters on the Pseudomonas chromosome. *tmexC6D6-toprJ1b* and several *tmexC6D6-toprJ1b*-like gene clusters exhibited distinct evolutionary differences from five previously characterized *tmexCD-toprJ* gene clusters and were more closely related to *tmexC3D5-toprJ2b*. Notably, the *tmexC6D6-toprJ1b*-like gene cluster existed on both chromosomes and plasmids, with Pseudomonas as the sole host bacterial species, whereas *tmexCD2-toprJ2*-like and *tmexCD3-toprJ1b*-like gene clusters were widely distributed in various bacterial species, including *Enterobacteriaceae* members, *Aeromonas*, and Pseudomonas (Fig. 1). These results confirm the emergence of several *tmexCD-toprJ* variants and suggest Pseudomonas as the major reservoir for them.

**FIG 2:**
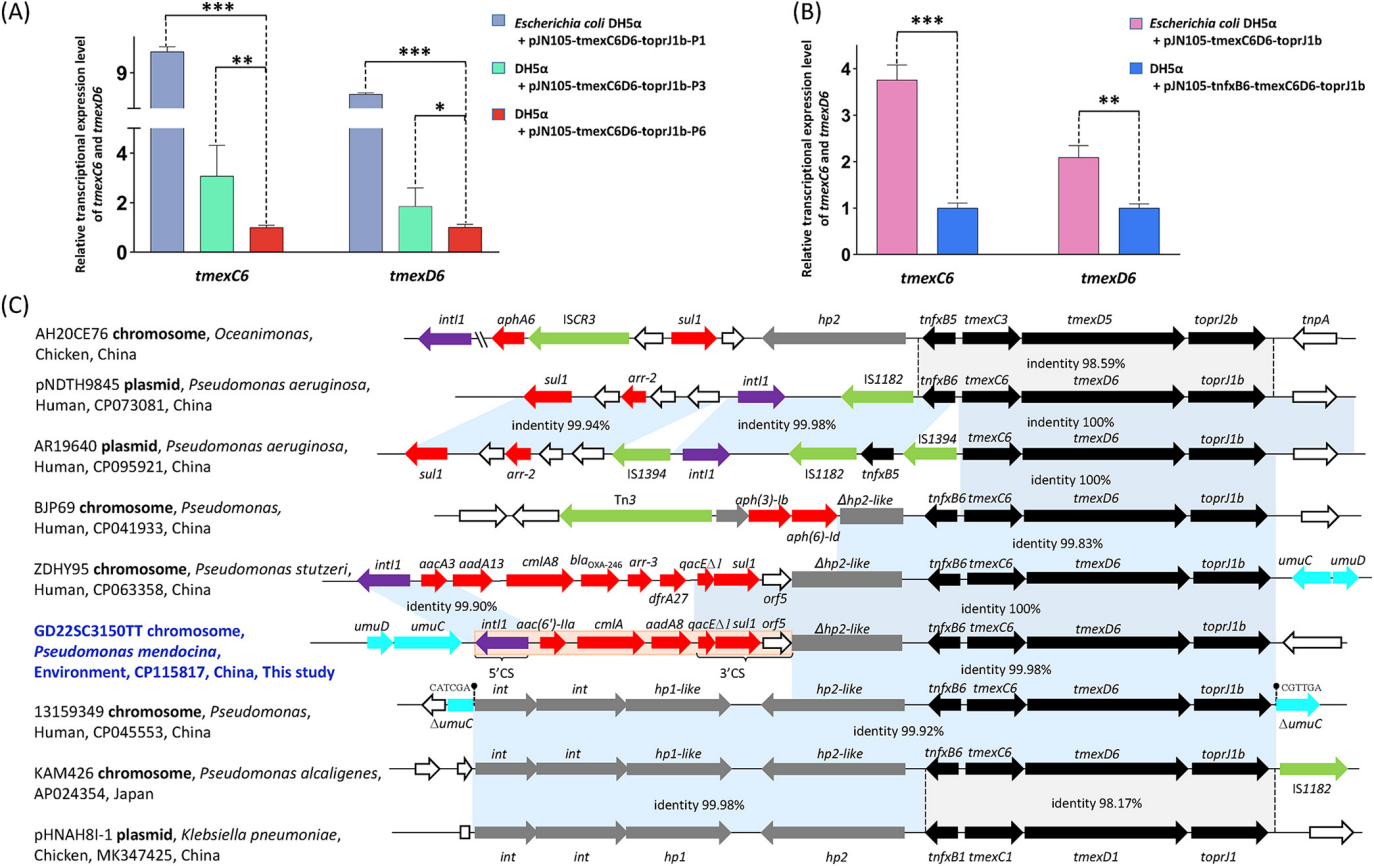
Regulation function and genetic characteristic of *tmexC6D6-toprJ1b*. (A) Transcriptional expression level of *tmexC6* and *tmexD6* in Escherichia coli recombinant strains carrying *tmexC6D6-toprJ1b* with three promoters. Levels for strains carrying P*_tmexC1_* and P*_tmexC3_* are shown relative to that of the strain carrying P*_tmexC6_*. (B) Transcriptional expression level of *tmexC6* and *tmexD6* in the presence of *tnfxB6* in recombinant strains relative to that in the absence of *tnfxB6*. 16S rRNA was used as the internal reference gene for quantitative reverse transcription-PCR to measure the relative mRNA fold changes. The level of statistical significance was analyzed by Student’s *t* test and shown as follows: ***, *P* values of <0.001; **, *P* values of <0.01; *, *P* values of <0.05. (C) Comparison of the genetic features of *tmexC6D6-toprJ1b* with those of its homologs. The extents and directions of genes are shown by arrows labeled by their names, and *tmexCD-toprJ* efflux pump genes are shown with black arrows, as well as various genes labeled with the according colors. Δ indicates a truncated gene, and horizontal lines represent the plasmid backbone. Gray shading indicates the nearly 100% identical sequence regions.

To investigate the function of *tmexC6D6-toprJ1b*, we constructed plasmids containing *tmexC6D6-toprJ1b* expressed with its native promoter (pJN105-tmexC6D6-toprJ1b-P6) using the primers listed in Table S3 and transformed them in Escherichia coli DH5α. Recombinant strains carrying *tmexC6D6-toprJ1b* showed an 8-fold increase in tigecycline MIC compared to that of the same bacteria carrying an empty vector (Table 1). Notably, the potential promoter region of *tmexC6D6-toprJ1b* (P*_tmexC6_*) showed three nucleotide differences from the promoters of *tmexC1* (P*_tmexC1_*) and *tmexC3* (P*_tmexC3_*). To investigate whether these promoter region differences influenced the function of *tmexC6D6-toprJ1b*, we replaced the promoter region of *tmexC6D6-toprJ1b* with P*_tmexC1_* and P*_tmexC3_*, thereby generating two vectors, pJN105-tmexC6D6-toprJ1b-P1 and pJN105-tmexC6D6-toprJ1b-P3, respectively. The expression of *tmexC6D6-toprJ1b* controlled by P*_tmexC1_* or P*_tmexC3_* increased the MICs of tetracyclines (4- to 32-fold), cefquinome (8-fold), streptomycin (4-fold), and ciprofloxacin (4-fold) relative to the same host strain carrying pJN105, while there was only a 2- to 8-fold increase of these agents with P*_tmexC6_* (Table 1). Meanwhile, different transcriptional expression levels of the *tmexC6D6-toprJ1b* gene cluster were observed in the three promoters, where strains with P*_tmexC1_* and P*_tmexC3_* exhibited significantly higher mRNA levels of efflux pump genes than those with P*_tmexC6_* (Fig. 2A). These results indicate that the promoter activity is critical for the efflux function of TMexC6D6-TOprJ1. To verify the potential regulatory effects of TNfxB6, *tnfxB6* was expressed accompanied by *tmexC6D6-toprJ1b* in the same pJN105 vector. A 4-fold decrease in tigecycline level was observed in the *tnfxB6-tmexC6D6-toprJ1b*-carrying strain relative to that in a strain without *tnfxB6* (Table 1). Moreover, the presence of TNfxB6 downregulated the transcriptional expression of the *tmexC6D6-toprJ1b* gene cluster (Fig. 2B), indicating the repressive effect of TNfxB6 on the expression of TMexC6D6-TOprJ1.

Unlike the previously discovered genetic structures of *tmexCD1-toprJ1* to *tmexCD3-toprJ1b* accompanied by an upstream integrase system (5, 7, 8), only a part of a hypothetical protein gene (*hp2*) was adjacent to the upstream region of *tmexC6D6-toprJ1b*. Notably, an integron carrying gene cassette *aac(6′)-IIa*/*aadA8*/*cmlA1* was inserted upstream of the *hp2* gene and formed a structure of 5′CS-*aac(6′)-IIa*-*aadA8*-*cmlA1-qacEΔ1-sul1-*3′CS-Δ*hp2-tnfxB6-tmexC6D6-toprJ1b* (Fig. 2C). Through BLASTN analysis, 16 additional *tnfxB6*-*tmexC6D6-toprJ1b* or *tnfxB6*-*tmexC6D6-toprJ1b*-like gene clusters (>99.8% nucleotide identity) were found in the plasmids or chromosomes of different Pseudomonas species from six countries: China, Pakistan, Poland, Lebanon, Spain, and Myanmar (Table S4). Interestingly, the upstream structure of a *tnfxB6*-*tmexC6D6-toprJ1b*-like gene cluster in Pseudomonas strain 13159349 was highly similar to that of the classical transferable module region (*int1-int2-hp1-hp2-tnfxB-tmexCD-toprJ*) of *tmexCD1-toprJ1* and was specifically inserted into the *umuC* gene (Fig. 2C).

In summary, we identified a novel multidrug resistance gene cluster, *tmexC6D6-toprJ1b*, which mediates tigecycline resistance, on the chromosome of Pseudomonas. Several *tmexCD-toprJ* variants are emerging in Pseudomonas, suggesting that Pseudomonas may be a major reservoir and ancestor of *tmexCD-toprJ*. Therefore, strong surveillance of these variants is necessary to prevent their uncontrolled spread.

### Data availability.

The complete sequence of the strain GD22SC3150TT chromosome was deposited in GenBank with the accession number CP115817.
