# Supplementary material for: Pseudomonas Acts as a Reservoir of Novel Tigecycline Resistance Efflux Pump tmexC6D6-toprJ1b and tmexCD-toprJ Variants
Source: Microbiol Spectr. 2023 Apr 17;11(3):e00767-23. doi: 10.1128/spectrum.00767-23 (PMC10269656; doi:10.1128/spectrum.00767-23)
Supplement: Supplemental file 1 — Supplemental material. Download spectrum.00767-23-s0001.pdf, PDF file, 0.2 MB [file spectrum.00767-23-s0001.pdf]

Supplementary Material

Table S1. Comparison of both the nucleotide and amino acid identity of *tnfxB6-tmexC6D6-toprJ1b* with its five homologs.

Gene cluster	Gene (protein)	*tnfxB6* (TNfxB6)	*tmexC6* (TMexC6)	*tmexD6* (TMexD6)	*toprJ1b* (TOprJ1)
TMO1	*tnfxB1* (TNfxB1)	97.67% (97.30%)			
	tmexC1 (TMexC1)		93.99% (97.16%)		
	tmexD1 (TMexD1)			96.27% (97.61%)	
	toprJ1 (TOprJ1)				99.93% (100.00%)
TMO2	*tnfxB2* (TNfxB2)	97.85% (97.84%)			
	tmexC2 (TMexC2)		94.59% (96.90%)		
	tmexD2 (TMexD2)			99.39% (99.90%)	
	toprJ2 (TOprJ2)				99.86% (99.79%)
TMO3	*tnfxB3* (TNfxB3)	99.82% (99.46%)			
	tmexC3 (TMexC3)		98.54% (98.45%)		
	tmexD3 (TMexD3)			96.84% (97.99%)	
	toprJ1b (TOprJ1)				100% (100%)
TMO4	*tmexC4* (TMexC4)		96.13% (97.16%)		
	tmexD4 (TMexD4)			96.40% (98.47%)	
	toprJ4 (TOprJ4)				90.17% (93.29%)
TMO5	*tnfxB5* (TNfxB5)	99.64% (98.92%)			
	tmexC3 (TMexC3)		98.54% (98.45%)		
	tmexD5 (TMexD5)			97.93% (99.04%)	
	toprJ2b (TOprJ2)				99.86% (99.79%)

Table S2 46 representative *tmexCD1-toprJ1*-like gene clusters used for building the phylogenetic tree.

GenBank accession number	Bacterial species	Isolation source	Isolation Country	Location of gene cluster
**This Study**	***Pseudomonas mendocina***	**Environment**	**China**	**Chromosome**
PRJNA877424	*Oceanimonas*	Animal	China	Chromosome
DADUYY010000124.1	*Escherichia coli*	Animal	Vietnam	Unknown
DAIVCG010000116.1	*Escherichia coli*	Animal	Thailand	Unknown
MT598646.1	*Pseudomonas aeruginosa*	Unknown	China	Plasmid
UOCO01000045.1	*Klebsiella pneumoniae*	Unknown	Unknown	Unknown
CP039832.1	*Aeromonas caviae*	Sewage/Environment	China	Chromosome
AP025277.1	*Aeromonas hydrophila*	Unknown	Vietnam	Chromosome
CP073356.1	*Proteus cibarius*	Animal	China	Chromosome
LLUU01000024.1	*Pseudomonas aeruginosa*	Human	Philippines	Unknown
MF344571.1	*Pseudomonas aeruginosa*	Unknown	China	Plasmid
MF344568.1	*Pseudomonas aeruginosa*	Unknown	Unknown	Plasmid
CP024630.1	*Pseudomonas aeruginosa*	Unknown	China	Chromosome
RHRM01000023.1	*Pseudomonas stutzer*	Environment	Pakistan	Unknown
JAEHID010000002.1	*Aeromonas caviae*	Human	China	Unknown
ABEUSQ010000072.1	*Pseudomonas aeruginosa*	Human	United States of America	Unknown
DAFQKW010000014.1	*Pseudomonas aeruginosa*	Human	Spain	Unknown
CP045553.1	*Pseudomonas*	Human	China	Chromosome
UIRA01000044.1	*Klebsiella pneumoniae*	Unknown	Unknown	Unknown
RHQZ01000017.1	*Pseudomonas stutzeri*	Environment	Pakistan	Unknown
CP035739.1	*Pseudomonas aeruginosa*	Human	Poland	Chromosome
CP064402.1	*Pseudomonas aeruginosa*	Human	China	Plasmid
CP043396.1	*Pseudomonas monteilii*	Drainage/Environment	China	Chromosome

CP087670	*Klebsiella pneumoniae*	Animal	China	Unknown
MZ532979.1	*Klebsiella pneumoniae*	Human	China	Plasmid
KU130294	*Pseudomonas putida*	Human	China	Plasmid
AP025011.1	*Raoultella ornithinolytica*	Unknown	Unknown	Plasmid
CP084031.1	*Aeromonas caviae*	Human	China	chromosome
MZ532981.1	*Klebsiella oxytoca*	Human	China	Plasmid
AAJFEK010000011.1	*Salmonella enterica*	Unknown	United States of America	Unknown
MN175502	*Raoultella ornithinolytica*	Human	China	Unknown
JAHQLM010000003.1	*Klebsiella quasipneumoniae*	Human	China	Unknown
CP091084.1	*Enterobacter roggenkampii*	Environment	China	Plasmid
JADTQG010000028.1	*Pseudomonas mendocina*	Human	United States of America	Chromosome
FNAE01000010.1	*Pseudomonas alcaliphila*	Unknown	United States of America	Chromosome
PGZI01000028.1	*Gammaproteobacteria bacterium*	Groundwater/Environment	Japan	Chromosome
CP070505.1	*Pseudomonas toyotomiensis*	Unknown	United Arab Emirates	Chromosome
NIQV01000010.1	*Pseudomonas toyotomiensis*	Environment	Japan	Chromosome
JACFYX020000004.1	*Pseudomonas*	Unknown	France	Chromosome
CP031606.1	*Pseudomonas*	Environment	Denmark	Chromosome
LT629797.1	*Pseudomonas sihuiensis*	Unknown	Unknown	Chromosome
AVQF01000014.1	*Pseudomonas chengduensis*	Stagnant water/Environment	India	Chromosome
JTFL01000132.1	*Pseudomonas oleovorans*	Unknown	China	Chromosome
JACETQ010000013.1	*Pseudomonas*	Seawater /Environment	Spain	Chromosome
QLYV01000114.1	*Pseudomonas*	Desert sand/Environment	Morocco	Chromosome
QLYU01000019.1	*Pseudomonas*	desert sand/Environment	Morocco	Chromosome

Table S3. Primers used in this study

Purpose	Primer name	Primer sequences
To detect *tmexC* gene	check-tmexC-F	TGAAGATCAACGCCGTCAGCC
	check-tmexC-R	ACATCACCAGGAACACCAGCAC
To construct pJN105-tmexC6D6-toprJ1b-P6	P6-tmexC-F	CTCATCGGCAGCAGTTAAATCCATC
	toprJ-R	CAATAATCCATAGGCCTGCCAGTC
	RE-pJN105-CDJ-F	GACTGGCAGGCCTATGGATTATTGGATCGCCCTTCCCAACAGTTGC
	RE-pJN105-CDJ-R	GATGGATTTAACTGCTGCCGATGAGGGAGAAACAGTAGAGAGTTGCGAT
To construct pJN105-tnfxB6-tmexC6D6-toprJ1b-P6	tnfxB6-F	GACACCGCCAACCCGTTGGAAAAAAACT
	toprJ-R	CAATAATCCATAGGCCTGCCAGTC
	RE-pJN105-nCDJ-F	GACTGGCAGGCCTATGGATTATTGGATCGCCCTTCCCAACAGTTGC
	RE-pJN105-nCDJ-R	TTTTCCAACGGGTTGGCGGTGTCGGAGAAACAGTAGAGAGTTGCGAT
To constructs pJN105-tmexC6D6-toprJ1b-P1 and pJN105-tmexC6D6-toprJ1b-P3	Promoter-C1-F	GAGTCAATATTGACTTATTTGATTTTTGCGTGGATCATGCG
	Promoter-C1-R	CGCATGATCCACGCAAAAATCAAATAAGTCAATATTGACTC
	Promoter-C3-F	ACATCAAAATCAAATGAGTTAATATTGACTCATTTGATTTTGGTGTGCATC
	Promoter-C3-R	GATGCACACCAAAATCAAATGAGTCAATATTAACTCATTTGATTTTGATGT
	RE-pJN105-F	CATTCAGGTCGAGGTGGCCC
	RE-pJN105-R	TACCTTGTCTGCCTCCCCG
To measure the transcriptional expression of tmexC6D6-toprJ1b and 16sRNA genes	qPCR-16srRNA-F	GCGTGGACTACCAGGGTATCTA
	qPCR-16srRNA-R	GTGTAGCGGTGAAATGCGTAGA
	qPCR-tmexC6-F	GGCGACGTGCTGTTCCAGAT
	qPCR-tmexC6-R	GGCTGACGGCGTTGATCTTCA
	qPCR-tmexD6-F	GTGCTGGTGTTCCTGGTGATGTT
	qPCR-tmexD6-R	CATGATCCGCTCGACGTTCTCC

Table S4. *tmexC6D6-toprJ1b* and *tmexC6D6-toprJ1b*-like gene clusters found in the GenBank

Nucleotide Identity compared with *tmexC6D6-toprJ1b*	Bacterial species	Location of gene cluster	GenBank accession number	Isolation source	Isolation Country
100%	*Pseudomonas stutzeri*	Chromosome	CP063358.1	Cerebrospinal fluid/Human	China
100%	*Pseudomonas stutzeri*	Chromosome	CP088004.1	Sediment/Environment	China
99.98%	*Pseudomonas*	Chromosome	CP045553.1	Abdominal fluid/Human	China
99.98%	*Pseudomonas aeruginosa*	Unknown	DAFQDY010000193.1	Human	Myanmar
99.83%	*Pseudomonas*	Chromosome	CP041933.1	Sputum specimen/Human	China
99.83%	*Pseudomonas*	Chromosome	CP113432.1	Activated sludge/Environment	China
99.83%	*Pseudomonas aeruginosa*	Plasmid	CP073081.1	Human	China
99.83%	*Pseudomonas aeruginosa*	Plasmid	CP095921.1	Rectal swab/Human	China
99.83%	*Pseudomonas aeruginosa*	Plasmid	CP064402.1	Human	China
99.83%	*Pseudomonas alcaligenes*	Chromosome	AP024354.1	Unknown	Unknown
99.83%	*Pseudomonas putida*	Chromosome	LR813085.1	Wastewater/Environment	Spain
99.83%	*Pseudomonas putida*	Chromosome	LR813083.1	Wastewater/Environment	Spain
99.81%	*Pseudomonas monteilii*	Chromosome	CP043396.1	Drainage/Environment	China
99.81%	*Pseudomonas monteilii*	Chromosome	CP043395.1	Drainage/Environment	China
99.81%	*Pseudomonadaceae*	Chromosome	CP113226.1	Pig/Animal	China
99.81%	*Pseudomonas aeruginosa*	Chromosome	CP061779.1	Urine/Human	Lebanon
99.81%	*Pseudomonas aeruginosa*	Chromosome	CP061777.1	Human	Lebanon
99.77%	*Pseudomonas aeruginosa*	Chromosome	CP035739.1	Eye/Human	Poland

99.77%	*Pseudomonas stutzeri*	Unknown	RHQZ01000017.1	Environment	Pakistan
